# Clinical epidemiology of non-alcoholic fatty liver disease in children and adolescents. The LiverKids: Study protocol

**DOI:** 10.1371/journal.pone.0286586

**Published:** 2023-10-13

**Authors:** Carla Chacón, Ingrid Arteaga, Alba Martínez-Escudé, Irene Ruiz Rojano, Noemí Lamonja-Vicente, Llorenç Caballeria, Ana María Ribatallada Diez, Helmut Schröder, Montserrat Montraveta, Maria Victoria Bovo, Pere Ginés, Guillem Pera, Galadriel Diez-Fadrique, Alba Pachón-Camacho, Núria Alonso, Isabel Graupera, Pere Torán-Monserrat, Carmen Expósito

**Affiliations:** 1 Unitat de Suport a la Recerca Metropolitana Nord (USR Metro-Nord), Institut Universitari d’Investigació en Atenció Primària Jordi Gol i Gurina (IDIAP Jordi Gol), Mataró, Barcelona, Spain; 2 Grup de Recerca en Malalties Hepàtiques a l’Atenció Primària (GRemHAp), IDIAP Jordi Gol, USR Metro-Nord, Mataró, Barcelona, Spain; 3 PhD Programme in Medicine and Translational Research, Faculty of Medicine, University of Barcelona, Barcelona, Spain; 4 Centre d’Atenció Primària Palaudàries, Institut Català de la Salut, Lliçà d’Amunt, Barcelona, Spain; 5 Centre d’Atenció Primària La Llagosta, Institut Català de la Salut, La Llagosta, Barcelona, Spain; 6 Centre d’Atenció Primària Dr. Barraquer, Institut Català de la Salut, Sant Adrià del Besos, Barcelona, Spain; 7 Centro de Investigación Biomédica en Red de Enfermedades Hepáticas y Digestivas (CIBEReHD), Barcelona, Spain; 8 Centre d’Atenció Primària Serraparera, Institut Català de la Salut, Cerdanyola del Vallès, Barcelona, Spain; 9 Hospital del Mar Medical Research Institute (IMIM), Barcelona, Spain; 10 CIBER Epidemiology and Public Health (CIBERESP), Instituto de Salud Carlos III, Madrid, Spain; 11 Paediatric Gastroenterology, Hepatology and Nutrition, Hospital Germans Trias i Pujol, Badalona, Spain; 12 Liver Unit, Hospital Clínic de Barcelona, School of Medicine and Health Sciences, University of Barcelona, Barcelona, Spain; 13 Institut d’Investigacions Biomediques August Pi I Sunyer (IDIBAPS), Barcelona, Spain; 14 Department of Endocrinology and Nutrition, Hospital Universitario Germans Trias I Pujol, Badalona, Barcelona, Spain; 15 Department of Medicine, Universitat Autònoma de Barcelona, Barcelona, Spain; 16 Center for Biomedical Research on Diabetes and Associated Metabolic diseases (CIBERDEM), Instituto de Salud Carlos III, Madrid, Spain; 17 Faculty of Medicine and Health Sciences, University of Barcelona, Barcelona, Spain; 18 Direcció d’Atenció Primària Metropolitana Nord Institut Català de Salut, Mataró, Spain; 19 Centre d’Atenció Primària Badia del Vallès, Institut Català de la Salut, Badia del Vallès, Barcelona, Spain; Universitatsklinikum Leipzig, GERMANY

## Abstract

**Background:**

Non-alcoholic fatty liver disease (NAFLD) is rapidly increasing alongside overweight and obesity, not only in adults but also in children and adolescents. It is unknown what impact the development of NAFLD in childhood may have in later life. The importance of early detection and treatment lies in its potential for progression to cirrhosis, liver cancer and liver-related death, as well as its associated extrahepatic comorbidities. Vibration-Controlled Transient Elastography (VCTE) with Controlled Attenuation Parameter (CAP) is an effective, non-invasive and safe diagnostic method to estimate the degree of fibrosis and steatosis in the liver, but little is known about its applicability in the paediatric population.

**Aims:**

1) To assess the prevalence of significant liver fibrosis (Liver Stiffness Measurement (LSM) ≥6.5 kPa) using VCTE, and that of non-alcoholic fatty liver disease (≥225 dB/m) using CAP in children and adolescents. 2) To determine the optimal cut-off points of the CAP to achieve maximum concordance with the Magnetic Resonance Imaging (MRI) findings in the diagnosis of mild, moderate and severe NAFLD in children and adolescents.

**Methods:**

Cross-sectional population-based study which will include 2,866 subjects aged between 9 and 16 years. Participants will undergo: anamnesis, physical examination, blood extraction, VCTE, MRI and questionnaires on socio-demographic data, personal and family medical history and lifestyle assessment.

**Applicability and relevance:**

The study aims to establish the foundations for the use of VCTE in children and adolescents in order to achieve early diagnosis of NAFLD. Moreover, it will serve to understand in further detail the disease and to identify the risk groups of children and adolescents who may be at risk of developing it. Ultimately, this will help determine to which subgroups of the population we need to target resources for prevention and early detection of this entity, as well as possible intervention for its treatment.

**Trial registration:**

The LiverKids study is registered on Clinicaltrials.gov (NCT05526274).

## Introduction

The increase of childhood obesity and its associated metabolic factors has led to an alarming rise in the incidence of non-alcoholic fatty liver disease (NAFLD) in this population. NAFLD is the accumulation of free fatty acids and triglycerides (TG) in the hepatocytes of individuals without toxic alcohol consumption and in the absence of other liver diseases. This pathology encompasses a wide spectrum of lesions ranging from simple steatosis to fibrosis, cirrhosis and even hepatocellular carcinoma [1].

NAFLD is the most common cause of liver disease and hypertransaminasaemia in the paediatric population, with a prevalence ranging from 7–9% and reaching 38–41% in obese children [2, 3]. Obesity is the main risk factor for NAFLD. The pathophysiological mechanisms are not yet fully understood, although the pathogenesis of NAFLD is considered to be multifactorial [4, 5]. The importance of early detection and appropriate management lies in its risk of progression to fibrosis and/or hepatocellular carcinoma along with its associated extrahepatic complications such as cardiovascular disease (CVD), diabetes mellitus (DM), insulin resistance (IR) and other endocrine and metabolic disorders like hypothyroidism or dyslipidaemia [6]. The presence of fibrosis is the major prognostic factor in NAFLD. Among children and adolescents with NAFLD, 25% have NASH and 14% have advanced fibrosis, the latter being more prevalent in paediatric patients with severe obesity than in adults with the same condition [7].

Diagnosis of NAFLD should be considered in paediatric population with excess weight, especially in those >10 years of age, with arterial hypertension (AHT), DM, IR or metabolic syndrome (MetS). In clinical practice, screening begins with anamnesis, physical examination, blood test and abdominal ultrasound, which help to establish a differential diagnosis with other entities. However, liver biopsy remains the gold standard for assessing disease progression, although its use is limited by its invasiveness and potential complications [8]. Some authors consider screening with transaminases, but these may still be normal in patients with NAFLD [5]. In terms of imaging, abdominal ultrasound, which is an affordable and safe technique, is only able to detect fatty infiltration above 30% [9]. Magnetic resonance imaging (MRI) is more accurate in diagnosing NAFLD, showing high sensitivity and specificity [10]. Nevertheless, its high cost and limited availability make it an invalid method for population screening.

Therefore, non-invasive serological and elastographic diagnostic methods are being developed. The most widely used is Vibration-Controlled Transient Elastography (VCTE), which non-invasively determines the presence of fibrosis by measuring liver stiffness (LSM) with the FibroScanⓇ (FS) device [11]. Multiple studies have demonstrated its usefulness in adults [11, 12], so it may also be appropriate for children and adolescents [5, 11]. Normal LSM values may vary according to age, ranging from 3.4–5.1 kPa [13, 14, 16]. Above this threshold, values correlate with different stages of fibrosis on the METAVIR scale: 5–7 kPa is indicative of F1; 7–9 kPa, F1-F2; and >9 kPa, F3-F4 [15, 16]. FibroScan features the Controlled Attenuation Parameter (CAP), which allows quantification of hepatic fat >11%. CAP values correlate with the degree of steatosis and a cut-off point of ≥225 dB/m is used for its detection [17, 18]. Furthermore, serological markers are used for diagnostic approaches to NAFLD and fibrosis. In adults, the Fatty Liver Index (FLI) [19] (NAFLD) as well as the NAFLD Fibrosis Score and FIB-4 (fibrosis) are commonly used [20] but these do not provide accurate results in the paediatric population [8, 21]. Thus, specific markers have been developed for these patients, such as the Pediatric NAFLD Fibrosis Index (PNFI) [22] and the Pediatric NAFLD Fibrosis Score (PNFS) [23], with an AUROC of 0.85 and 0.74 respectively [22, 23].

Ultimately, the only effective approach to prevent the progression of NAFLD and its associated comorbidities consists of lifestyle changes. Some studies have shown histological improvement after weight loss [24]. At present, no specific diet or physical exercise has been shown to improve the progression of NAFLD beyond the benefit of weight loss. Moreover, obesity and NAFLD tend to start developing at an early age and, in most cases, this development continues into adulthood with the hepatic, metabolic and cardiovascular consequences that this entails. Considering that any treatment is less effective in the late stages (F3-F4) of the disease compared to the early stages (F1-F2), screening for NAFLD as well as investigating the lifestyles and risk factors that may be related to it, especially from an early age, is key to its prevention and early management; this could have a very important health, social and economic impact [25].

## Study rationale and hypotheses

In recent years, large epidemiological studies are being conducted to screen for liver fibrosis in the adult population using non-invasive methods. These studies are showing alarming results regarding the increase of people with NAFLD mainly associated with overweight and obesity [12, 26]. It is known that children and adolescents with NAFLD are at greater risk of developing NASH and fibrosis in adulthood. Yet, there are no strategies for early detection of this entity among this population. In fact, the only studies performed using the CAP for the detection of NAFLD in children and adolescents, to date, have an extremely limited sample size, are conducted either in population at risk or with underlying liver disease, or their results are compared with non-accurate methods such as abdominal ultrasound [17, 27–30]. Hence, the main hypotheses stated in this study are: 1) Screening NAFLD using a non-invasive method such as VTCE in children and adolescents may be useful in detecting asymptomatic subjects with significant liver steatosis and fibrosis; and 2) The degree of NAFLD can be estimated by the CAP in a form analogous to that of MRI in children and adolescents.

Early detection of NAFLD in children and adolescents is key for its appropriate management and the prevention of its progression and that of its associated hepatic and extrahepatic complications. This would allow: a) to validate VCTE as a simple and safe screening method to advance in the early diagnosis of NAFLD in a highly vulnerable population such as paediatrics; and b) to determine to which subgroups of the infant-juvenile population we need to direct resources for the prevention and early detection NAFLD or any of its complications as well as the possible intervention for their treatment. Ultimately, all of this would allow the implementation of strategies aimed at halting NAFLD progression, thus preventing the development of severe related clinical outcomes in later life, which would result in adding additional years of healthy life.

### Study aims

The study main aims are: 1) To assess the prevalence of significant liver fibrosis (LSM ≥6.5 kPa) using VCTE, and that of NAFLD (≥225 dB/m) using the CAP in children and adolescents; and 2) To determine the optimal cut-off points of the CAP to achieve maximum concordance with the MRI findings in the diagnosis of mild, moderate and severe liver steatosis in children and adolescents.

### Study design

The present study has been registered on Clinicaltrials.Gov (NCT05526274) on 1^st^ September 2022. Inclusion of the first participant in the study took place in November 2022.

This is a cross-sectional and population-based study that will include 2,866 subjects in Catalonia (Spain). Eligible participants are being invited to join the study through informative talks held in their own educational centres. In these informative talks, for potential participants, their parents and their teachers, the date of the first visit is being scheduled and the socio-demographic data and medical history questionnaire are being distributed for participants to bring back completed on the day of Visit 1.

- Visit 1 (V1): is carried out on all subjects, in the nurses’ offices of the educational centres. V1 includes: anamnesis, physical examination, VCTE to assess LSM and CAP, and collection of the completed socio-demographic and medical history questionnaire. After V1, all subjects are scheduled for Visit 2 and Visit 3. In addition, subjects with altered results (CAP ≥225 dB/m and/or LSM ≥ 6.5kPa) and 10% of subjects with normal results are referred to Visit 4. Lastly, subjects with suspected significant fibrosis (LSM ≥6.5 kPa) are referred to the Digestive Service of the Hospital Universitari Germans Trias i Pujol (HUGTP) for further evaluation (Visit 5).
- Visit 2 (V2): is conducted at the educational centres and consists on lifestyle assessment (Diet Quality estimation with the KIDMED index [31]), physical activity (PA) assessment with the Physical Activity Unified-7 item Screener (PAU-7S) [32], and sedentary behaviour [33], sleep duration [34], and sugar consumption assessment) through online self-reported questionnaires with the assistance of trained personnel.
- Visit 3 (V3): is performed in the Primary Care Centres corresponding to each subject. In V3, all subjects undergo blood sampling. The samples are analysed in the HUGTP laboratory.
- Visit 4 (V4): is performed in the Diagnostic Imaging Centres located within the area of the educational centres. MRIs are performed on all subjects with CAP ≥225 dB/m and/or LSM ≥6.5 kPa and 10% of participants with CAP <225 dB and/or LSM <6.5 kPa.
- Visit 5 (V5): is performed in the Digestive Service of the HUGTP. Subjects with suspected significant fibrosis (LSM ≥6.5 kPa) undergo the corresponding diagnostic tests and follow-up according the Clinical Practice Guideline for the Diagnosis and Treatment of NAFLD in Children [25].

**Fig 1:**
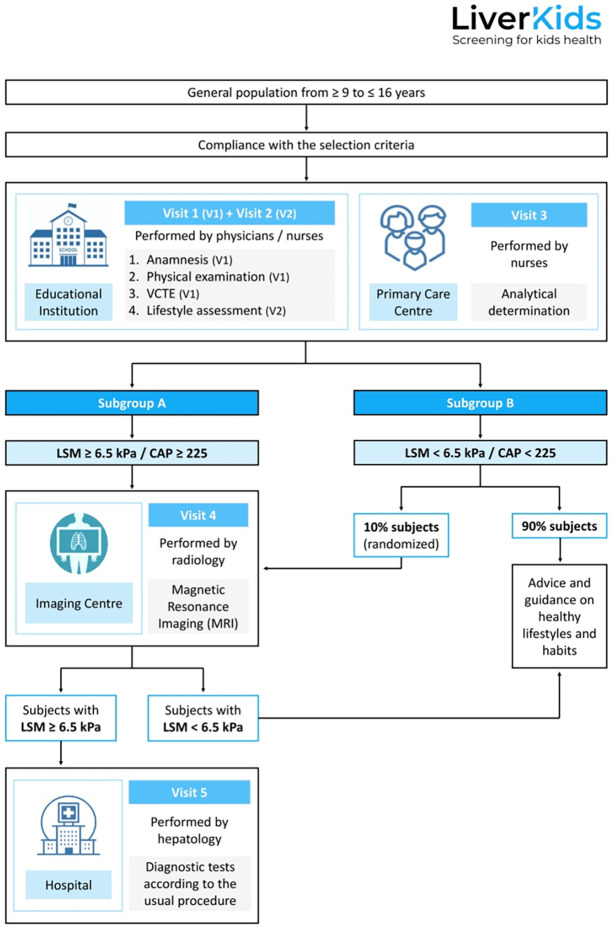
Study procedure flow chart. VCTE: Vibration-Controlled Transient Elastography; LSM: Liver Stiffness Measurement; CAP: Controlled Attenuation Parameter.

Finally, all subjects (whether they obtain CAP ≥225 dB/m and/or LSM ≥6.5 kPa or CAP <225 dB/m and/or LSM <6.5 kPa), and especially those with NAFLD risk factors such as obesity, DM, MetS or risky alcohol consumption are counselled on lifestyle modifications by the health care professionals involved in the study [35]. They are also referred to their corresponding healthcare providers for management of these risk factors according to each centre’s own protocol (Fig 1).

### Main outcome measures

1. Percentage of subjects with LSM ≥6.5 kPa by VCTE in the general population aged ≥9 to ≤16 years.
2. Percentage of subjects with CAP ≥225 dB/m by VCTE in the general population aged ≥9 to ≤16 years.
3. Comparison of liver steatosis diagnosis accuracy between CAP by VCTE and MRI in the general population aged ≥9 to ≤16 years.
4. Determination of optimal cut-off points of the CAP for the diagnosis of mild, moderate and severe liver steatosis in children and adolescents.

### Specific outcome measures

1. Comparison of liver steatosis diagnosis accuracy between CAP by VCTE and Fatty Liver Index (FLI) in the general population aged ≥9 to ≤16 years.
2. Comparison of liver fibrosis diagnosis accuracy between LSM by VCTE and two fibrosis scores (Pediatric NAFLD Fibrosis Index and Pediatric NAFLD Fibrosis Score) in the general population aged ≥9 to ≤16 years.
3. Detection and structuring of the variables that constitute a specific biomarker for the paediatric-juvenile population that is capable of accurately determining the presence of hepatic steatosis.
4. Correlation of liver steatosis diagnosis using CAP by VCTE, FLI and MRI, with the presence of lifestyles and socio-economic factors associated with NAFLD in the general population aged ≥9 to ≤16 years.
5. Percentage of subjects with BMI ≥30 in the general population aged ≥9 to ≤16 years.
6. Percentage of subjects with MetS according to the International Diabetes Federation (IDF) consensus criteria for children and adolescents [36] in the general population aged ≥9 to ≤16 years.

### Recruitment and enrollment of participants

Subjects aged between 9 and 16 years who are enrolled in the compulsory education system in the territory of Mataró (Catalonia). Eligible patients have been identified following the EU General Data Protection Regulation (GDPR-2018) and then randomised and stratified by schools and institutes. These educational centres have a social class value (high/middle/low) assigned according to the MEDEA variable [37] and are differentiated by type (charter/private or public). A number of educational centres have been randomly selected from each social class-type combination, taking into account the number of students enrolled in each educational centre in order to reach the necessary sample that is representative of the students attending school in Mataró.

General practitioners and primary care nurses invite subjects to participate in the study from schools. Informative talks are held at the educational centres in which the legal guardians of the subjects are informed about the study and are requested to give their written informed consent for their children’s participation. Once subjects and/or their legal guardians accept to participate in the study, they are scheduled a visit at their own educational center where they are informed about the details of the study and enrolled if they meet the selection criteria outlined below.

- *Inclusion criteria*: subjects between 9 and 16 years of age whose legal guardians have provided written informed consent for their children’s participation in the study. Subjects over 12 years of age are also required to give their own written informed consent.
- *Exclusion criteria*: subjects with previously diagnosed liver disease, receiving hepatotoxic drug treatment, with eating disorders diagnosed prior to study entry and/or children/adolescents who do not attend class on the day of the measurements.

### Premature withdrawal

All subjects are free to withdraw from the study at any time. Investigators may also decide, for reasons of clinical safety, to withdraw subjects from the study. The date and justification for discontinuing the study is recorded in the patient’s medical record, in the patient’s data collection form and in the corresponding database.

### Data collection and measuring tools

Data are being collected from November 2022 to June 2023, in educational centres, primary care centres and diagnostic imaging centres. Organisation of data collection follows the procedure outlined in Table 1.

**Table 1:** Organisation of data collection procedures.

	Visit 1	Visit 2	Visit 3	Visit 4	Visit 5
	At educational centres	At primary care centres	At educational centres	At Diagnostic Imaging Centres	At the HUGTIP Hospital
Selection criteria					
Informed Consent					
Socio-demographic data					
Anamnesis and clinical history					
Physical examination					
Analytical determination					
Lifestyle assessment					
VCTE					
MRI					
Diagnostic tests / follow-up according to usual clinical procedure					

VCTE, Vibration-Controlled Transient Elastography; MRI, Magnetic resonance imaging.

**Socio-demographic data** include: age, sex, household income, parents’ education level, educational centre.

**Anamnesis and clinical history** include:

- Comorbidities and pathological history: AHT, DM, dyslipidaemia, obesity or any other chronic diseases.
- Alcohol consumption: in standard drinking units (SDU).
- Tobacco consumption.
- Regular use of medication and/or other toxics.
- Family clinical history: family history of obesity, NAFLD, AHT, DM, dyslipidaemia and CVD.

**Lifestyle assessment** is self-reported online at the participating schools with the assistance of trained personnel.

- Diet Quality estimation: KIDMED index [31] is used. It is based on 16 items with a dichotomous (yes/no) response format, and estimates adherence to the Mediterranean diet in children/adolescents. The index ranges from -4 to 12, and is based on a 16-question test. Answers are assigned a value of -1 or +1, respectively. The sums of these values are classified into 3 levels: a) >8, optimal diet; b) 4–7, improvement needed; c) <3, very low diet quality.
- PA assessment: PAU-7S [32] is used. PAU-7S consists of 7 questions referring to the week before the test. It takes different types of PA into consideration (walking; team sports; individual activity), as well as the context where it was practiced (schoolyard; after school and on weekends; physical education classes). The last question refers to whether the child was ill during the previous week or was unable to perform PA for some reason. Questions 1–6 are answered according to 5 possible options that refer to time spent (no activity, 0 minutes; <30 minutes; 30–60 minutes; 60–90 minutes; and >90 minutes. The questionnaire gives the total amount of minutes dedicated to PA in the week before the test is taken.
- Other child/adolescent lifestyle variables:
  - Sedentary behaviour is assessed by the Screen-time Sedentary Behaviour Questionnaire [33], which asks about time spent in: 1) watching TV, 2) playing computer games, 3) playing video games, 4) using mobile phone, separately for weekdays/weekends.
  - Sleep duration is recorded by 4 questions on hours of sleep from the Sleep Habits Survey for Adolescents that ask about bedtime and time of waking up on weekdays/weekends [34].
  - Consumption of sugar containing carbonated/no-carbonated beverages is assessed by standardized questions.

**Physical examination** is carried out by healthcare workers (minimum of 2) with participants in their underwear and barefoot.

- Anthropometric data: weight (kg), height (cm), Body Mass Index (BMI) [38–40], waist circumference (WC) [40] and thoracic circumference.
- Tanner’s pubertal stage: it classifies and divides pubertal changes into five successive stages, ranging from infant (grade I) to adult (grade V).
- Determination of blood pressure.
- **Analytical determination** is performed on all subjects, after 12 hours of fasting, at the Primary Care Centre. It includes:
- Blood count, glucose, glycosylated haemoglobin, basal insulin levels, total cholesterol, high-density lipoprotein (HDL), low-density lipoprotein (LDL), triglycerides (TG), aspartate transaminase (AST), alanine transaminase (ALT), gamma-glutamyl-transpeptidase (GGT), alkaline phosphatase (ALP), total bilirubin, sideremia, transferrin, transferrin saturation index, total protein, albumin, urea, creatinine, sodium, potassium, thyroid stimulating hormone (TSH) and tiroxine (T4).
- Determination of insulin resistance using the HOMA method: blood glucose (mmol/L) x insulin (mU/L)/22,5.

**Metabolic Syndrome (MetS)** is determined according to the IDF consensus criteria for children and adolescents [36]. These include a WC at the ≥90th percentile and the presence of 2 or more of the following variables: 1) TG ≥150 mg/dl, HDL <40 mg/dl. 2) Blood pressure ≥130/85 mmHg. 3) Blood glucose ≥100 mg/dl or previously diagnosed DM.

**Liver fibrosis** is estimated by two methods: LSM and liver fibrosis scores.

- LSM is performed in all patients using VCTE with the Fibroscan® (FS) system (Echosens, France). LSM is performed with S, M or XL probe following the Automatic Probe Selection tool available on the FS. The FS used is the 430 MINI. FS measures the hepatic elasticity of a cylinder approximately 1 cm in diameter by 4 cm in length [41]. FS transmits, via a probe, a vibration that propagates through the liver. The speed of propagation of the vibration correlates with the stiffness (in kPa) of the liver tissue and allows estimation of the fibrosis degree. In order for VCTE to be valid, 10 correct determinations with an interquartile range (IQR) <30 and a success rate >60% must be obtained. Significant liver fibrosis by VCTE is being defined by a LSM ≥6.5 kPa [13–16].
- Fibrosis scores/serum biomarkers: Pediatric NAFLD Fibrosis Index (PNFI) and Pediatric NAFLD Fibrosis Score (PNFS) are being used to estimate fibrosis degree. Significant liver fibrosis is being defined when the values of the different scores are above the following cut-offs, according to literature: PNFI ≥9, PNFS ≥13 [22, 23, 42, 43].

**Liver steatosis** is estimated using three methods: CAP, liver steatosis score and MRI.

1. CAP using VCTE is used to measure liver steatosis with the FS using either S, M or XL probe at the time of LSM. Liver steatosis is diagnosed when the value of CAP ≥225 dB/m [13–16].
2. Liver steatosis is measured by the steatosis score Fatty Liver Index (FLI) which is based on: WC, BMI, TG and GGT [19]. Significant liver steatosis is defined when FLI ≥60 according to literature [44].
3. Liver steatosis measured by MRI. It is performed using the Magneton Essenza 1.5 Tesla Aera system (SIEMENS), with the integration of Tim technology and Dot Engines (Day Optimizing through put Engines) application packages. The result obtained is the steatosis score or steatosis degree corresponding to the percentage of hepatocytes containing fatty vacuoles with respect to the hepatic fat fraction. Thus, a differentiation is made among mild steatosis (5–29% of hepatocytes affected), moderate steatosis (30–59% affected) and severe steatosis (more than 60% of hepatocytes affected). MRI is performed on all participants with altered VCTE results (≥6.5 kPa and/or ≥225 CAP) and 10% of participants with normal VCTE results (<6.5 kPa or <225 CAP).

### Sample size calculation

The school population aged between 9 and 16 in Mataró stands at 11,503 individuals [45]. These are approximately equally distributed between the 4 groups: male/female between 9 to 12 and 13 to 16 years old.

We expect that 8% of this population will have NAFLD and 2% will have liver fibrosis. A sample size of 2,866 students will allow us to estimate a prevalence of NAFLD stratified by age (9–12, 13–16 years) and sex of 8%, with a precision of 2% in each stratum, and a prevalence of fibrosis of 2%, with a precision of 1% in each stratum. We anticipate that blood samples will be available in 50% of the sample and MRI in 70%. MRI will be offered to all LSM/CAP positive subjects (LSM ≥6.5 kPa / CAP ≥225 dB/m) and, randomly, to 10% of those with negative LSM/CAP (LSM <6.5 kPa / CAP <225 dB/m). This will add up to 1,433 blood tests and 424 MRIs.

With the proposed sample we can find a concordance of 80% with a precision of 4% using kappa indices, or an intraclass correlation coefficient of 0,8 with a precision of 2,5% (8% and 4% precision for kappa and ICC, respectively, using only 424 MRIs). This sample size has been calculated assuming a design effect of 20%, an alpha error of 5%, a power of 80% and bilateral contrasts.

### Statistical analyses

1. Prevalences of NAFLD and fibrosis will be calculated using CAP ≥225dB/m and LSM ≥6.5kPa, respectively. They will be calculated by sex and age (9–12 and 13–16 years) and 95% confidence intervals will be provided.
2. To determine the optimal CAP cut-off point that best fits the 4 MRI categories, the relationship between CAP and MRI will be explored graphically, CAP means for each MRI category will be compared by ANOVA, 2-to-2 ROC curves will be used, and a multi-class ROC curve will be used to obtain the overall area under the curve for the finally chosen points. In addition, dichotomous CAP classifications (normal/altered) will be compared with MRI levels using contingency tables and chi-square tests.
3. To identify the variables related to NAFLD, multivariate models will be used using NAFLD as the dependent variable and possible risk and confounding factors as independent variables.
4. Concordance between the diagnosis of NAFLD or fibrosis obtained by different methods (CAP vs FLI, LSM vs PNFI, LSM vs PNFS) will be assessed by kappa indices (when using the dichotomised version of these variables) and by the intraclass correlation coefficient (when using the continuous version of the variables). The best cut-off point in PNFI and PNFS will be chosen to detect fibrosis using LSM ≥6.5 kPa as the standard test in the diagnosis of fibrosis using ROC curves.
5. The construction of the biomarker to detect the presence of steatosis in children and adolescents will be carried out using the coefficients of multivariate logistic regression models, where steatosis (CAP >225 dB/m) will be the dependent variable and the rest of the variables will be potential explanatory variables that may form part of the marker.

To take into account that participants come from selected schools, hierarchical models will be used when necessary. All tests will be bilateral, with a significance of 5%. We do not plan to use missing data imputation methods as we expect very few missings beyond those already considered. Stata17 statistical package will be used.

### Risks and disadvantages for patients

There are minimal disadvantages for the subjects arising from their participation in this study. In fact, the only ones expected are the following: 1) Blood collection may be accompanied by discomfort or temporary swelling or bruising at the puncture site. All possible measures will be taken to minimise this effect. 2) MRI is not painful, non-invasive and does not use radiation, but the patient must remain in an enclosed machine, which may be a problem for claustrophobic patients. However we will explain that it is a completely harmless and safe procedure, that in no case will any type of medication, contrast or sedation be used, and that it is an opportunity to obtain health related relevant information. In the event that MRI is still not well tolerated by the patient, we will disregard it and offer it to another eligible subject. Meanwhile, VCTE is well tolerated and the procedure does not represent any potential risk for the patient or adverse effects. Thus, overall, the benefits of participating in this study outweigh the risks.

### Ethics

The study is being conducted in accordance with the Declaration of Helsinki, agreed at the 64th General Assembly in 2013. Confidentiality and anonymity of the data will be ensured in accordance with current state laws (Organic Law 3/2018, of 5 December, on Personal Data Protection and guarantee of digital rights) both in the execution phase and in the presentations or publications arising from the study. All researchers in direct contact with participants must strictly comply with the Law on the Protection of Children and Adolescents, passed in August 2015.

This project has been approved by the Ethics and Clinical Research Committee of the IDIAP Jordi Gol (protocol code 22/096-P, first date of approval 27/04/2022; date of approval of amendments 14/12/2022) for studies involving humans, it will be under its supervision during its development and will incorporate its recommendations. Written informed consent from the legal guardians of all subjects is requested for the participation of their minor (<18 years of age) children in the study. In addition, for subjects over 12 years of age, their own written informed consent to participate in the study is also required.

As mentioned above, the benefits of participating in this study outweigh the risks. There is no specific treatment for NAFLD. The only approach that has been shown to be effective in preventing the progression and comorbidities associated with NAFLD is based on lifestyle changes and weight loss, which would improve the progression and metabolic profile of these patients. Therefore, lifestyle modification interventions can be implemented in patients diagnosed with chronic liver disease due to NAFLD. Patients with risk factors for NAFLD but without liver steatosis or fibrosis yet will also be given advice on healthy lifestyles in order to prevent its future development.

## Expected results

As regards the direct results related to the main objectives of this project, it is expected to be obtained that: 1) the prevalence of liver fibrosis is around 2% and that of NAFLD is around 8% among the general population aged 9–16 years; 2) the degree of NAFLD can be estimated by the CAP in a form analogous to that of MRI in children and adolescents.

The project will provide essential information on childhood NAFLD. That is particularly relevant as we are at a time when the burden of chronic liver diseases is on the rise and, moreover, it is expected to increase in the coming years. The results of this project with the exhaustive tests will allow (1) to assess the current prevalence of both NAFLD and liver fibrosis in the general population between 9 and 16 years, (2) to understand in further detail the comorbidities associated with childhood obesity and to identify the risk groups of children and adolescents who, without being obese, may be at risk of developing NAFLD or any of its complications and (3) the validation of VCTE as a non-invasive, simple and safe method to progress in the early diagnosis of chronic liver diseases in an easy and safe way in a highly vulnerable population such as paediatrics. Hence, this project will allow the development of strategies and algorithms adapted to the infant-juvenile population, enabling the early diagnosis of NAFLD from a much earlier age.

In addition, the LiverKids study will serve as a basis from which to generate a population cohort that can be followed in the long term to obtain more information on the evolutionary course of NAFLD from childhood onwards.

## Study status

Enrolment of the first participant in the study took place in November 2022 and recruitment is expected to be completed by June 2023.

### Discussion

The burden of NAFLD is expected to increase alarmingly in the coming years alongside the surge in overweight and obesity. We do not know the potential impact that the development of NAFLD in children and adolescents may have in the adult stage. Furthermore, it is known that children and adolescents with NAFLD are at greater risk of developing NASH and fibrosis in adulthood. This is critical because fibrosis is the major prognostic determinant of the disease. Although there is no specific medical treatment for NAFLD, weight loss has been shown to be effective; in fact, it can induce NASH resolution and fibrosis improvement by at least one stage. However, treatment is less effective in the later stages of the disease than in the earlier ones. Even so, there are no strategies for early detection of this entity.

Therefore, there is a pressing need to shift the present approach from diagnosing NAFLD in adulthood, when it may have already progressed to NASH and/or fibrosis or led to extrahepatic complications, to diagnosing the disease sooner, when complications have not yet arisen. Moreover, the impact of lifestyle interventions in adult life is limited. Nevertheless, children and adolescents do show positive results in learning and integrating healthy lifestyle habits.

Overall, early diagnosis and management will be the only way to tackle the looming epidemic of NAFLD. To date, no population-based study has been conducted to assess the prevalence of NAFLD in children and adolescents or the long-term impact it may have. The LiverKids is a pioneering study that constitutes one of the first steps in addressing this pathology, which is why its implementation will have a very significant social, health and economic impact.

## Conclusions

The LiverKids study will allow us not only to assess the current prevalence of both NAFLD and liver fibrosis in the infant-juvenile population but to understand in further detail the comorbidities associated with childhood obesity and to identify the risk groups of children and adolescents who, without being obese, may be at risk of developing NAFLD or any of its complications. Thus, once these factors have been established, we will be able to determine to which subgroups of the population we need to direct resources for the prevention and early detection of these entities as well as the possible intervention for their appropriate management. In addition, the validation of VCTE as a non-invasive, simple and safe method will allow to progress in the early diagnosis of chronic liver diseases in an easy and safe way in a highly vulnerable population such as paediatrics.

Framing NAFLD screening as a challenge for preventive practices will give us the opportunity to avoid the development of the advanced stages of the disease and its severe outcomes such as liver cirrhosis, hepatocarcinoma and/or liver-related death, as well as its multiple associated extrahepatic comorbidities.

## Supporting information

S1 File Data protection and management. (PDF)

S2 File Information sheet and consent for participants. (PDF)

S3 File Information sheet and consent participants’ legal guardians. (PDF)

## Abbreviations

AHT: Arterial Hypertension
ALP: Alkaline Phosphatase
ALT: Alanine Transaminase
AST: Aspartate Transaminase
BMI: Body Mass Index
CAP: Controlled Attenuation Parameter
CVD: Cardiovascular Disease
dB/m: Decibels per Meter
DM: Diabetes Mellitus
FLI: Fatty Liver Index
FS: Fibroscan
GGT: Gamma-Glutamyl-Transpeptidase
HDL: High-Density Lipoprotein
HUGTP: Hospital Universitari Germans Trias i Pujol
IR: Insulin Resistance
kPa: Kilopascals
LDL: Low-Density Lipoprotein
LSM: Liver Stiffness Measurement
MetS: Metabolic Syndrome
MRI: Magnetic Resonance Imaging
NAFLD: Non-Alcoholic Fatty Liver Disease
NASH: Non-Alcoholic Steatohepatitis
PA: Physical Activity
PNFI: Pediatric NAFLD Fibrosis Index
PNFS: Pediatric NAFLD Fibrosis Score
SDU: Standard drinking units
T4: Tiroxine
TG: Triglycerides
TSH: Thyroid Stimulating Hormone
VCTE: Vibration-Controlled Transient Elastography
WC: Waist Circumference
