# Supplementary material for: Clinical epidemiology of non-alcoholic fatty liver disease in children and adolescents. The LiverKids: Study protocol
Source: PLoS One. 2023 Oct 13;18(10):e0286586. doi: 10.1371/journal.pone.0286586 (PMC10575486; doi:10.1371/journal.pone.0286586)
Supplement: S1 File — (PDF) [file pone.0286586.s001.pdf]

DATA PROTECTION AND MANAGEMENT

Below, we outline how data relating to each of the following points will be processed:

1) Identification of the data and the subjects who process them.

All data will be processed in a pseudo-anonymised way by coding the identifying variables in both the initial paper Data Collection Form (DCF) and the subsequently created database. Only the necessary data to achieve the research objectives will be collected. The variables needed to conduct the study are the following: 1) socio-demographic data, 2) personal and family history, 3) habits and lifestyles, 4) physical examination, 5) blood determination (Blood count, glucose, glycosylated haemoglobin, basal insulin levels, total cholesterol, high-density lipoprotein (HDL-cholesterol), low-density lipoprotein (LDL-cholesterol), triglycerides, transaminases (AST, ALT), gamma-glutamyl-transpeptidase (GGT), alkaline phosphatase, total bilirubin, sideremia, transferrin, transferrin saturation index, total protein, albumin, urea, creatinine, sodium, TSH and T4) , 6) transient elastography and 6) magnetic resonance imaging. These variables can be consulted in the corresponding section of the methodology. All this information, which will be recorded in the DCF (Annex 1), will be obtained both from the questionnaire carried out on the participants and from the analytical determinations, elastographies and MRI scans also performed on the participants. In accordance with Law 14/2007, the samples of each participant in the project will be collected directly from the person concerned and the procedure set out in the regulations on the use of biological samples will be followed to authorise their use. The samples will only be used for the LiverKids project and will subsequently be destroyed. The data will be kept for 15 years.

The "Institut Català de la Salut" (ICS) and the "Institut Universitari d'Investigació en Atenció Primària" (IDIAP Jordi Gol) will act as data controllers in the framework of this observational study. The IDIAP Jordi Gol will be in charge of data processing. The database will be deposited in an ICS server located at the "Unitat de Suport a la Recerca Metropolitana Nord" (USR Metro-Nord) that complies with security regulations and will act as a data processor. Researchers accessing the archive will have to use a password.

Each participant will have an identifier code for the study. Thus, the PI of the study will have a dissociated folder containing the data relating the identifier code with the CIP of each participant. Participants and their parents/legal guardians will sign the informed consent to give permission to access their medical history data. The study investigators will only have access to patient codes and patient-related information as data processors. The ICS will have access to the questionnaire and the clinical information obtained during the visits.

The data controller will store the pseudo-anonymised data and subjects will only be re-identified for the sole and exclusive purpose of conducting the LiverKids study, having adopted specific security measures to prevent access by unauthorised third parties.

2) Identification and legitimate basis for processing.

The necessary variables to perform the study will be obtained directly from the participants in the project by means of a self-completed form to be filled in by the participant and his/her legal guardian. The exploratory variables, biological samples and complementary explorations

will be obtained by accredited personnel. All variables and data obtained will require the prior consent of the participant and, in the case of children under 12 years of age, also that of their parents/legal guardians, in accordance with the provisions of articles 6.1.a) and 9.2.a) of the RGPD, as well as additional provision 17.2.d of the LOPD-GDD. The pseudo-anonymisation technique to be used will be the cryptographic hash function.

3) Tools used for data processing.

The RedCap platform and a project-specific database will be used to carry out the project. The database will be created and stored on an ICS server that complies with the security measures determined by the institution.

The centre in charge of performing the MRI scans (GEMA/CETIR) will be responsible for processing and transmitting the data to the ICS and IDIAP Jordi Gol professionals in charge of the study. For this purpose, the corresponding contract will be signed with this entity. The data transmitted from GEMA/CETIR to ICS will be transmitted encrypted, only with a code and without any personal identifiers. All data will be related to a code and only ICS, IDIAPJGol and GEMA/CETIR professionals linked to the study will be in charge of processing these data.

The project data will be only stored on ICS servers where the organisation has installed the software and therefore only accessible on computers that have a trusted connection via VPN and secure credentials (certificates, RSA keys or complex passwords). These computers will follow the security standards set by the ICS in compliance with current requirements.

4) International data transfers.

International data transfers are not foreseen. In the event of such transfers, they would only be to countries that guarantee adequate compliance with data protection regulations due to the existence of an adequacy decision or any other legally authorised mechanism.

5) Identification of processing operations that may pose a high risk to the rights and freedoms of participants in the research project.

In accordance with the provisions of Article 35 of the RGPD, this project does not meet the necessary characteristics that require the corresponding impact assessment to be carried out.

6) Content of the information sheet and informed consent of participants and their parents/legal guardians in the research project.

- Both the information sheet and the informed consent of participants and parents/legal guardians refer to how the data will be protected.
- The informed consent of both parents and participants states that participation in the study involves undergoing a blood test. In addition, it includes a specific item of acceptance for the performance of the blood test.
- Both the information sheet and the informed consent of participants and parents/legal guardians state the right of participants and/or parents/legal guardians to withdraw their consent to the processing of such data, at any time, by sending an e-mail to the address cexposito.mn.ics@gencat.cat.
