# Supplementary material for: Clinical epidemiology of non-alcoholic fatty liver disease in children and adolescents. The LiverKids: Study protocol
Source: PLoS One. 2023 Oct 13;18(10):e0286586. doi: 10.1371/journal.pone.0286586 (PMC10575486; doi:10.1371/journal.pone.0286586)
Supplement: S2 File — (PDF) [file pone.0286586.s002.pdf]

PARTICIPANT INFORMATION SHEET

Study title: Clinical epidemiology of non-alcoholic fatty liver disease in children and adolescents. LiverKids study.

Abbreviated title: Non-alcoholic fatty liver disease in children and adolescents.

Protocol code: LiverKids.

Sponsor: Institut Universitari d'Investigació en l'Atenció Primaria Jordi Gol i Gurina (IDIAP Jordi Gol), Barcelona, España.

Principal Investigator: Carmen Expósito Martínez

Site: ……………………………………………………………………………….................….

- **Invitation**

We invite you to participate in a research study called the LiverKids study. In this study we are assessing, using a non-invasive screening method, the prevalence of non-alcoholic fatty liver disease and liver fibrosis in children and adolescents.

Before you decide whether you are willing to participate, it is important that you understand why this study is being conducted. Please take the time to read this information sheet and discuss it with your friends, family or doctor. Also, don't hesitate to ask questions if you are unclear about anything or would like more information.

- **What is the aim of the study?**

The main objective of the study is to assess the prevalence of non-alcoholic fatty liver disease and liver fibrosis in children and adolescents using the FibroScan®. FibroScan is a non-invasive, simple and widely used method that measures both liver stiffness and liver fat and identifies fibrosis in the liver.

Overweight and obesity have been increasing rapidly in recent years. In turn, diseases related to weight gain have been appearing and one of them is non-alcoholic fatty liver disease (NAFLD). NAFLD is characterised by the presence of fat in the liver tissue of people who neither have high alcohol consumption nor other liver diseases. Although the disease generally has a benign course, a significant percentage of patients may develop more severe forms of the disease such as fibrosis, cirrhosis or even liver cancer. In addition, NAFLD is an important risk factor for the development of other diseases such as pre-diabetes, diabetes, insulin resistance, dyslipidaemia and cardiovascular disease. NAFLD is currently the most common liver disease in Western countries in both children and adults and is expected to increase along with overweight and obesity.

Liver cirrhosis is the final stage of all chronic liver diseases and is associated with high morbidity and mortality. Chronic damage to the liver causes inflammation and fibrosis and the progressive accumulation of fibrosis over a period of 20-30 years leads to cirrhosis. In general, patients are not diagnosed during the developmental stage of fibrosis as they have no symptoms and do not need medical attention. The main factor predicting the long-term outcome of patients with chronic liver disease is the existence of liver fibrosis. Yet there are no strategies for early detection of NAFLD or fibrosis. This is important because, to date, there is no specific treatment for this disease. As the disease is closely related to obesity, only lifestyle changes such as diet and exercise have proven to be effective as they act directly on weight loss. Furthermore, treatments are less effective in the late stages compared to the early stages of the disease.

Standard liver tests used to assess liver function are not accurate methods to detect NAFLD and fibrosis. In fact, at present, definitive diagnosis is only established by liver biopsy, a costly, invasive and risky procedure. For this reason, further research is increasingly being conducted into methods that are just as effective as biopsy, but less invasive. In recent years, a new non-invasive method has been developed to assess the presence and severity of liver fibrosis, liver elastography using the FibroScan® device. This method is based on the measurement of liver stiffness. Liver elastography is a technique that can be used directly in primary care centres and can be performed in 5-10 minutes, without pain or sedation. Furthermore, the new FibroScan devices are associated with a new technology that also allows to assess the amount of fat in the liver. Thus, this technique seems suitable for the early detection of chronic liver diseases in children and adolescents.

In our country we do not know the percentage of children and adolescents with NAFLD, nor do we have sufficient data on the impact it has on the infant-juvenile population. We also do not know the consequences that this may entail in adulthood. The present study will allow us to know the current prevalence of both NAFLD and liver fibrosis in children and adolescents and to identify their associated risk factors. Thus, once these factors have been established, we will be able to determine to

which subgroup of the population we should direct resources for the prevention and early detection of these entities and possible intervention for their treatment.

- **Why have I been invited to participate?**

You have been invited to participate in this study because it is a population-based study in which participants are randomly selected from the general population. You have been invited to participate randomly, not because you have a risk factor for liver disease.

- **Do I have to participate in this study?**

No. Your participation in this study is completely voluntary. If you decide to participate, you are free to withdraw from the study at any time without giving any reason. Your decision to withdraw from the study or not to participate will not affect the care you receive on a regular basis from your doctors and nurses.

Samples and data collected during your participation in the study will be stored and analysed, unless you request their destruction. However, it would not be possible to return your samples or remove your data from the study results if they have already been processed at the time you decide to withdraw them from the study.

- **What will happen if I participate in the study?**

If you participate in this study, you and your parents/legal guardians will be asked to sign the informed consent form provided to you.

A total of 2,866 participants from different schools in the Barcelona (Spain) will take part in this study. The study will have a total duration of 3 years. Your participation in the study will not last longer than 3 months during which you may be asked to attend a diagnostic imaging centre on 1 occasion depending on the result obtained at the first visit.

If you decide to participate in the study and are eligible to do so, you will be given a questionnaire about your medical history, family background and lifestyle habits to complete at home with your parents/legal guardians and bring it with you to the first visit. A first visit will take place at your school. At this visit, a nurse will review your questionnaire with you, perform a physical examination including vital signs and perform a FibroScan® to measure liver stiffness. All of this will take no more than 15 minutes. To perform the test, you will lie face up on a stretcher and the probe with the transducer will be placed on your skin in the area where the right hepatic lobe is located. In addition, a complete blood test will be performed at your primary care health centre. The total amount of blood that will be drawn at this visit will be about 21 mL.

If the results of the first FibroScan® visit are normal, you may be randomly offered to undergo a magnetic resonance imaging (MRI) scan at a diagnostic imaging centre in your city. Once the MRI has been performed, or after the first visit has been completed, your participation in the study will be terminated. If, on the other hand, the FibroScan® results suggest that you may have significant liver fibrosis (≥6.5Kpa) and/or high steatosis values (≥225 CAP), you will automatically be offered an MRI scan. Moreover, if the FibroScan® results suggest that you may have significant liver fibrosis (≥6.5Kpa), we will refer you to the third level referral hospital (Hospital Germans Trias i Pujol) to continue with the diagnostic tests according to the usual procedure in order to confirm the diagnosis of liver disease and evaluate its origin.

- **What are the possible disadvantages of participating in the study?**

Blood collection may be accompanied by discomfort or temporary bruising at the puncture site. All possible measures will be taken to minimise this effect.

The FibroScan® is a device with a transmitter-receiver probe that is placed between the ribs at the level of the liver and emits a short, low-frequency mechanical wave (50Hz). This vibration wave is transmitted into the liver tissue at a speed that depends on the stiffness of the tissue, the stiffer the tissue, the faster the vibration. The speed is detected by ultrasonography by the probe itself and the device's software transforms the speed (m/s) into an elasticity value (kilopascals or kPa). The FibroScan® takes about 10 minutes and is well tolerated. To obtain a reliable liver elasticity value, 10 valid measurements must be taken. The procedure does not represent any potential risk for the patient or any adverse effects.

MRI is a test that uses powerful magnets and radio waves to create images of the body. No ionising radiation (x-rays) is used to perform the test. You will be asked to wear a hospital gown or clothing without metal fasteners (such as sweatpants and a

T-shirt). Certain types of metal can cause blurred images. You will then lie on a table, which slides into a large tunnel-like tube. During the MRI, the person operating the machine will watch you from another room. The test lasts approximately 30 to 60 minutes. The MRI involves no potential risk of side effects.

- **What are the potential benefits of participating in the study?**

After the tests have been carried out, we will inform you of your specific situation. If you are found to have chronic liver disease with fibrosis, you will benefit from an early diagnosis and can therefore be offered treatment for your disease. This treatment will be carried out according to the recommendations of internationally accepted Clinical Practice Guidelines and will be exactly the same as if you were not participating in the study. Additionally, the information obtained as a result of this study could help us to improve the early detection of chronic liver disease in children and adolescents.

- **Expenses and compensation**

There is no cost to you for participating in the study. You will not receive any compensation for participating in this study.

- **What happens if there are any problems?**

If you have any questions about any aspect of this study, you can contact the study investigators who will do their best to answer your questions. You will find their contact details on this information sheet.

- **Will my participation in this study be kept confidential?**

Yes, all information collected about you during the study will be kept strictly confidential. Once the information is collected, it will be transferred to a database where an encrypted identification code will be created and no information that would allow your identity to be recognised will be recorded.

The "Institut Català de la Salut" (ICS) and the "Institut Universitari d'Investigació en Atenció Primària" (IDIAP Jordi Gol) will act as data processors in the framework of this study. The database will be deposited in an ICS server located in the "Unitat de Suport a la Recerca Metropolitana Nord" (USR Metro-Nord) which complies with security standards and will act as data processor. Researchers accessing the archive will have to use a password.

The centre in charge of performing the MRI scans (GEMA/CETIR) will be responsible for processing and transmitting the data to the ICS and IDIAP Jordi Gol professionals in charge of the study. For this purpose, the corresponding contract will be signed with this entity. The data transmitted from GEMA/CETIR to the ICS will be transmitted encrypted, only with a code and without personal identifiers. All data will be linked to a code and only ICS, IDIAPJGol and GEMA/CETIR professionals linked to the study will be able to process these data.

The project data will only be stored on ICS servers where the organisation has installed the software and will therefore only be accessible on computers that have a trusted connection via VPN and secure credentials (certificates, RSA keys or complex passwords). These computers shall follow the security standards established by the ICS that comply with the current requirements.

Thus, any study information about you that comes out of the primary care centre or hospital will not contain your name and address and you will not be recognised. Only persons directly involved in the study are authorised to access participants' names. All data collected for the study that can identify you will be kept confidential. This data will be identified by a code and only the study doctors/nurses will be able to link this data to you and your medical history. If the results of this study are published, your identity will be kept confidential.

The processing of your personal data will comply with the provisions of Organic Law 3/2018 of 5 September on the Protection of Personal Data and Guarantee of Digital Rights and Regulation (EU) 2016/679 on the protection of individuals with regard to the processing of personal data and on the free movement of such data. In accordance with the provisions of the aforementioned regulation, you may exercise your rights of access, rectification, erasure, restriction of processing, portability and opposition to processing. It is also important for you to know that this proposal has been reviewed and approved by the Research Ethics Committee of the IDIAP Jordi Gol.

- **What will happen with my samples?**

The blood samples obtained during the study will be analysed in the laboratory of the reference hospital (Hospital Germans Trias i Pujol). Any information on the blood samples that could identify you will be removed. Your samples will be identified by a study code. All samples obtained will be used solely for the purposes of this study. After analysis, the samples will be destroyed in compliance with Law 14/2007 on Biomedical Research.

- **What will happen with the study results?**

The results of the study will be presented at international research conferences and published in research journals. The results will be reported in an internal clinical study report that will be sent to the Ethics Committee. You will not be identified in this report or in any presentation or publication.

- **Who organises and funds the research?**

This is a non-commercial research study designed and developed by the Grupo de Investigación en Enfermedades Hepáticas en la Atención Primaria (GRemHAp). The GRemHAp is a group accredited as a consolidated group and expert in conducting population-based studies. The sponsor of the study is the IDIAP Jordi Gol (Barcelona, Spain).

- **Who reviewed the study?**

This study has been reviewed and approved by the IDIAP Jordi Gol Ethics Committee. Furthermore, the study will be conducted in accordance with the Declaration of Helsinki.

- **What happens if I decide not to take part in the study?**

You may withdraw from the study at any time, but information that has been collected up to the time of withdrawal may be used.

CONTACT DETAILS FOR FURTHER INFORMATION

Name of the Principal Investigator: Dr. Carmen Expósito Martínez

Study coordinator: Carla Chacón

Contact telephone number: 93 741 53 38

Thank you for reading this information sheet.

INFORMED CONSENT OF THE PARTICIPANT

Study title: Clinical epidemiology of non-alcoholic fatty liver disease in children and adolescents. LiverKids study.

Abbreviated title: Non-alcoholic fatty liver disease in children and adolescents.

Protocol code: LiverKids.

Sponsor: Institut Universitari d'Investigació en l'Atenció Primaria Jordi Gol i Gurina (IDIAP Jordi Gol), Barcelona, España.

Principal Investigator: Carmen Expósito Martínez

Site: ……………………………………………………………………………………………………

1. I confirm that I have read the information sheet for the above study. I have had the opportunity to consider the information, ask questions and have had my questions answered to my satisfaction.
2. I understand that my participation is voluntary and that I am free to withdraw at any time without giving any reason and without affecting my legal rights or my medical treatment.
3. I understand that my participation in the study involves the collection of a blood sample by means of a blood test.
4. I freely agree to participate in this study and consent to the access and use of my data under the conditions detailed in the patient information sheet.
5. I wish to exercise my right to be informed of the results obtained with the studies carried out on my samples, including when the findings have a significant implication on my health or that of my family members.

   Yes ☐ No ☐

6. I agree to be contacted in the future by members of the study research team to obtain information related to my health status.

   Yes ☐ No ☐

7. I agree to participate in the above-mentioned study.

Participant's name	Date	Signature

Investigator's name (principal or collaborator)	Date	Signature

Two copies must be signed, one for the participant and one to be filed in the medical record.
